# A Study on Online Gaming and Its Effect on Emotional Regulation – Online Survey

**DOI:** 10.1192/bjo.2025.10160

**Published:** 2025-06-20

**Authors:** Poornima Khadanga, Madhavi Rairikar, Gaurav Pawar, Gomati Nadadgalli

**Affiliations:** 1 Northamptonshire Healthcare Foundation NHS Trust, Northampton, United Kingdom; 2 Salvation Church Medical Centre, Mumbai, India; 3 Symbiosis Medical College for Women, Pune, India; 4 KLE’s JGMMC, Hubballi, India

## Abstract

**Aims:** The study aimed to study the prevalence of problematic online gaming and find out association between problematic gaming with domains of emotional regulation.

**Methods:** An online survey questionnaire was created by the researchers after approval and validation from experts and sent through various social media platforms. Those who did not consent for the study were excluded. The questionnaire included basic sociodemographic details and questions taken from two scales – (i) Problematic Online Gaming Questionnaire SF for identifying problematic online gaming and (ii) Difficulties in Emotional Regulation Scale (DERS-18) to identify domains of emotional regulation. Descriptive statistics, Pearson correlation, ANOVA were used for statistical analysis.

**Results:** 108 participants reverted with the completed forms, out of which, 69.4% were regularly engaged in online gaming, 19.4% fell under the domain of problematic online gaming. 4.6% of the participants were engaged in online gaming for more than 8 hours in a day. With regards to various domains of emotional regulation, the mean scores in each subscale of emotional regulation (Nonacceptance of Emotional Responses, Difficulties Engaging in Goal-Directed Behaviour, Impulse Control Difficulties, Lack of Emotional Awareness, Limited Access to Emotion Regulation Strategies and Lack of Emotional Clarity) were more in those with problematic online gaming as compared with non-problematic individuals and was statistically significant (p<0.05). There was also a positive correlation between problematic online gaming and sub-domains of emotional regulation (p<0.01).

**Conclusion:** The study clearly depicts the rise in online gaming that has been clearly demonstrated in the recent studies worldwide. The results clearly illustrate the association of online gaming with difficulty in handling emotions but also a means to attenuate negative emotions. It could be potentially being used as an escape mechanism. Though details of the causal link between the two parameters were beyond the scope of the study, it would be worth looking at it in future research.

